# Clinical Characteristics and Genetic Variants in Children with *PAX2* Mutation-Associated Disorders

**DOI:** 10.3390/medicina61060959

**Published:** 2025-05-22

**Authors:** Yanyan Jin, Na Li, Zipei Chen, Ke Zeng, Jingjing Wang, Aiqin Sheng, Haidong Fu, Lidan Hu, Jianhua Mao

**Affiliations:** 1 Department of Nephrology, The Children’s Hospital, Zhejiang University School of Medicine, National Clinical Research Center for Child Health, Hangzhou 310052, China; 2 School of Medicine, Zhejiang University, Hangzhou 310027, China; 3 Department of Pediatric, The First Affiliated Hospital of Ningbo University, Ningbo 315211, China

**Keywords:** *PAX2*, genotypes, clinical phenotypes, gene sequence

## Abstract

*Background and Objectives:* PAX2 serves as a critical transcription factor integral to the process of embryogenesis. Variations in the *PAX2* gene could result in the aberrant development of numerous organs. Despite the identification of numerous mutations within the *PAX2* gene, the correlation between specific genotypes has yet to be fully clarified. The objective of this study was to examine the clinical phenotypes and genotypes associated with *PAX2* mutation-induced disorders in pediatric patients of Chinese descent. The aim of our study was to forecast the pathogenic potential of these genetic mutations and to ascertain possible correlations between genotypic variations and the clinical manifestations of disorders linked to *PAX2* mutations. *Materials and Methods:* We recruited 14 pediatric subjects with *PAX2* mutations, meticulously examining the clinical characteristics and genetic alterations present in these individuals. Computational techniques were utilized to evaluate the pathogenicity, stability, and biophysical characteristics. A range of computational tools were employed for this assessment, including PredictSNP, MAGPIE, iStable, Align GVGD, ConSurf, and SNP effect. *Results:* The age at onset ranged from prenatal to 12 years. Five patients progressed to end-stage renal disease. Proteinuria and bilateral renal hypoplasia were observed in 92% of cases. Ocular and auditory abnormalities were also noted. We identified eleven different *PAX2* mutations, including five novel variants not previously reported in the literature. We predicted that all mutations, with the exception of p.F27-L33 del and N188S, exhibited high pathogenicity scores. In particular, R117P and R140W are strongly associated with disease pathogenicity and are likely to cause more significant damage than other gene mutants. *Conclusions:* This study expands the mutational and phenotypic spectrum of *PAX2*-related disorders in the pediatric population. The identification of five novel variants enhances our understanding of the genetic basis of these conditions. Despite recurrent mutations, marked phenotypic heterogeneity persists, underscoring the need for further research.

## 1. Introduction

*PAX2* (paired box gene 2, OMIM #167409) is an important transcription factor involved in embryonic development. It plays a significant role in regulating gene transcription during the development of various organs, such as the kidneys, eyes, ears, and central nervous system [1,2]. *PAX2* is located at 10q24.31 and spans approximately 80 kb. It consists of 12 exons. The PAX2 protein contains a highly conserved DNA-binding pairing domain, which functions as a nuclear transcription factor by binding to DNA [3]. During the embryonic stage, it is widely expressed in the renal duct epithelium and mesenchyme and plays a crucial role in the development of nephrotic cords, the formation and elongation of renal ducts, the branching of ureteral buds, and the initiation of nephron formation [4]. It can inhibit apoptosis and facilitate mesenchymal–epithelial transdifferentiation (MET). Patients with homozygous mutations in *PAX2* typically experience bilateral kidney agenesis and severe lung hypoplasia, which often leads to death shortly after birth. In contrast, patients with heterozygous mutations in *PAX2* exhibit a range of clinical manifestations. These include renal coloboma syndrome (RCS), oligogiant nephronopathy, bilateral renal hypodysplasia, congenital renal and urinary tract malformations (CAKUT), and focal segmental glomerulosclerosis type 7 (FSGS7) [5]. According to previous studies, 92% of patients with heterozygous *PAX2* mutations have structural or functional abnormalities of the kidneys, and 100% of those with renal hypodysplasia are likely to develop end-stage renal disease (ESRD) [6,7]. Unfortunately, there is currently no effective treatment for this condition. Many patients begin renal replacement therapy at a certain point in their lives and eventually undergo kidney transplantation. In addition to renal manifestations, previous reports indicate that approximately 77% of individuals have ocular abnormalities, with the most common being optic nerve colobomas. These two syndromes are collectively referred to as renal coloboma syndromes. Other conditions include high-frequency hearing loss (affecting 7% of individuals), congenital heart disease, and skeletal deformities [6].

*PAX2*-related mutations have been reported with 375 gene mutations (LOVD database, www.lovd.nl/PAX2, retrieved on 22 February 2025). However, these diseases exhibit diverse clinical manifestations, involve various organs, and cause various degrees of damage to renal function. There appears to be no exact correspondence between clinical phenotypes and genotypes, and the exact pathogenic mechanism and treatment methods remain unclear. In this study, we examined 14 children who were diagnosed with *PAX2* mutation-related diseases at the Department of Nephrology, Children’s Hospital of Zhejiang University. We thoroughly recorded and compared their clinical characteristics and conducted a detailed analysis of their genotypes and variant pathogenicity. We found a significant proportion of novel variants and interesting phenomena, such as variable penetrance. Our study aimed to improve the clinical diagnosis of *PAX2*-related diseases while exploring potential pathogenic mechanisms.

## 2. Materials and Methods

### 2.1. Samples

From October 2018 to October 2024, a total of 14 children with *PAX2* mutation-related diseases were recruited at the Children’s Hospital of Zhejiang University (2023-IRB-0319). These children received a comprehensive clinical assessment and underwent whole-exome genetic testing (WES) due to either renal dysplasia or unexplained renal failure. Disease confirmation was based on a combination of clinical phenotypes, genetic variants, and pathogenicity analysis. Children with *PAX2* variants whose pathogenicity could not be determined or did not match the clinical phenotype were excluded from the study. Informed consent was obtained from all parents, who provided authorization for the use of their medical records and genetic testing data.

### 2.2. Clinical Assessment and Ancillary Examinations

In this study, we collected comprehensive data on these 14 children when they first visited our hospital, including general information, demographic characteristics, medical details, laboratory tests, and imaging tests. We evaluated indicators related to the urinary system, including kidney function, urine tests, and urinary system structure. Additionally, we assessed the presence of abnormalities in commonly involved extrarenal organs, including the eyes, hearing, and nervous system. Finally, we tracked the most recent renal function assessment and age of progression to end-stage renal disease (ESRD). The corrected 24 h creatinine clearance rate was used as the estimated glomerular filtration rate (eGFR) for the 11 children whose 24 h urine was successfully collected. For the remaining 2 children who were younger than 1 year old, we calculated the eGFR via the Schwartz formula (eGFR = K height/serum creatinine), with a K value of 40.

### 2.3. Whole-Exome Genetic Testing

After providing informed consent, we collected EDTA-anticoagulated peripheral blood from parents and children for whole-exome genetic testing (WES). Furthermore, Sanger verification was conducted on the parents at the corresponding mutation sites for cosegregation analysis. Whole-exome sequencing (WES) was performed for all patients and their parents to detect potential disease-causing *PAX2* variants. Identified variants were cross-referenced with major databases (HGMD, gnomAD, ClinVar) and classified according to the 2019 ACMG guidelines [8].

### 2.4. Pathogenicity, Stability, and Biophysical Prediction In Silico

We assessed the pathogenicity, stability, and biophysical properties of the 5 missense mutations that were likely pathogenic and of uncertain significance (see Section 3.3) via in silico methods. To evaluate these parameters, we utilized tools such as PredictSNP, iStable, MAGPIE, and Align GVGD. PredictSNP, a consensus classifier, integrates multiple algorithms, such as MAPP, PhD-SNP, PolyPhen1, PolyPhen2, SIFT, SNAP, PANTHER, and the nsSNP analyzer [9]. These tools provide valuable information about the nature of mutations, distinguishing between deleterious and neutral variants. iStable uses an SVM to calculate the impact of an SNP on protein stability. By combining predictions from I-Mutant 2.0, MUpro, and iStable [10], integration enhances predictive power, surpassing that of individual tools. Align GVGD, a free online tool, integrates protein multiple sequence alignment (MSA) and the biophysical features of amino acids to predict the position of a nsSNP on a scale ranging from enriched neutral to enriched deleterious. Aligning GVGD extends the use of the Grantham difference to simultaneous multiple comparisons and MSA. Inputting amino acid sequences and mutations yields class assignments ranging from class 15 (considered neutral) to class 65 (considered deleterious) [11]. Mutants identified as deleterious, impacting stable, or altering biophysical properties were further assessed for their degree of evolutionary conservation via the ConSurf web server (v 1.05) [12]. The MAGPIE server (v 0.1.0) was utilized to predict and analyze the pathogenicity of multiple variants in gene sequences. This tool requires the gene sequence and specific mutation as inputs, after which it generates a number from 0 to 1, where 0 means benign mutation and 1 means highly pathogenic mutation [13]. To predict and analyze the structural effects of point mutations on protein sequences, the HOPE server (v 2010) was employed. HOPE takes the protein sequence and mutation as input and generates a comprehensive report, including results, animations, and figures.

## 3. Results

### 3.1. General Characteristics

Among the 14 children included in the study, 4 were boys, and 10 were girls (1:2.5). The onset age of the children ranged from prenatal to 12 years. Five cases, including patients 1, 2, 3, 4, and 5, progressed to ESRD, with the youngest case being only 1 year old. The reasons for medical consultation varied among the cases, with renal impairment present in six cases (patients 1, 2, 3, 4, 14, and 6), proteinuria in four cases (patients 8, 11, 12, and 5), urinary system structural abnormalities in two cases (patients 10 and 13) detected during prenatal examination, nocturnal enuresis in one case (patient 9), and renal cyst in one case (patient 7).

### 3.2. Clinical Characteristics

#### 3.2.1. Renal Manifestations

Upon initial examination, the serum creatinine (SCr) levels of thirteen children exhibited varying degrees of elevation (92%). The renal function of five children was classified as Chronic Kidney Disease (CKD) stage 5, that of five children as CKD stage 3, and that of the remaining four children as CKD stages 1–2. A urine examination indicated proteinuria in ten children (71%). Twenty-four-hour urine protein quantification revealed that six children had nephrotic-range proteinuria (≥50 mg/kg/d), and five out of these six (83%) had progressed to End-Stage Renal Disease (ESRD). Microscopic hematuria was detected in two children (HBC ≥ 3/HP). Symptoms indicative of renal involvement, including proteinuria, small kidneys, or elevated creatinine levels, were noted in thirteen patients (excluding Patient 13, 92%) [14]. Renal imaging, comprising ultrasound and MRI, disclosed bilateral renal hypodysplasia in thirteen children (92%), renal cysts in five children (35%), unilateral renal agenesis in one child (7%), and urinary tract obstruction in two children (14%). Among the five children with renal cysts, three had multiple small cysts in both kidneys, and two had a solitary cyst in one kidney. All cysts were less than 0.5 cm in diameter. Detailed information is available in Table 1.

#### 3.2.2. Extrarenal Manifestations

For the evaluation of extrarenal organs, we assessed the commonly involved eyes, hearing, nervous system, and bones. Among them, three of the five children who underwent ophthalmological evaluation had abnormalities (3/5, 60%), including two cases of papilledema and one case of bilateral morning glory fundus. Seven children underwent a hearing test, with only one case failing the otoacoustic emission test (1/7, 14%). Upon evaluating the nervous system and development, no obvious delays in language or motor development were observed in any of the patients. However, two patients suffered from short stature, and one patient experienced enuresis. In terms of bones, we also found new phenotypes involving scoliosis in two patients. Further details are provided in Table 2.

### 3.3. PAX2 Mutations

#### 3.3.1. Variants Report

Eleven mutation types have been reported in 14 children, with five mutation types not previously documented (LOVD and ClinVar, retrieved on 22 February 2025). The gene variants are detailed in Table 3. Seventy-eight percent (10/14) of the mutations were located in or encompassed the paired domain (exons 2–4). In terms of mutation types, there were five cases of frameshift mutations, five cases of missense mutations, and one case each of nonsense mutations, CNV deletions, and splice mutations. Cosegregation analysis revealed that ten patients had de novo mutations, whereas the remaining four inherited the variant from their parents. However, only two of them had a family history, suggesting differences in the penetrance of the gene. According to the AGMD rules, among the eleven variants, nine were identified as pathogenic/possibly pathogenic (P/LP), and two were of uncertain pathogenicity (VUS). Neither our pediatric cohort nor previously reported cases show a clear correlation between phenotype and genotype. Even among patients with the same mutation, such as patient No. 2/7/8/9, who all had the c.76dupG mutation, there were differences in their degree of renal function damage, urinary protein amount, renal imaging characteristics, and extrarenal manifestations. This suggests that there may be other pathogenic mechanisms that have not yet been identified.

**Figure 1:**
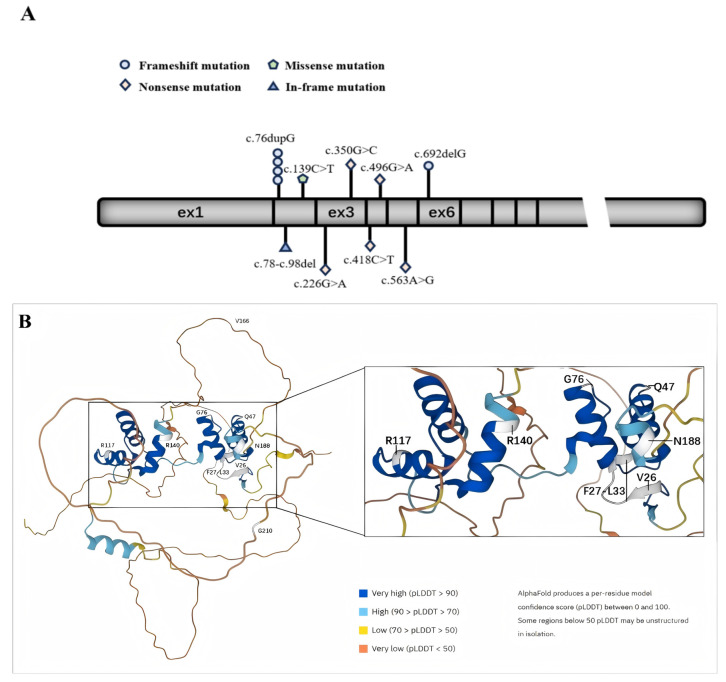
Schematic diagram of the exon structure and protein structure of *PAX2*. (**A**) The exon structure of *PAX2*; (**B**) protein structure of *PAX2*.

In Figure 1, we plot the relative positions of gene variants on *PAX2* and the relative positions of amino acid variants on a schematic protein structure generated by Alphafold website.

In picture A, the geometric shapes indicate the relative positions of different types of mutations. Picture B shows a schematic diagram of the protein structure produced by Alphafold. The white area shows the position of the mutated amino acid.

#### 3.3.2. Pathogenicity and Stability Prediction Using In Silico Tools

A total of five mutations were classified as either deleterious or neutral by PredictSNP. Among the individual algorithms used by PredictSNP, MAPP, PhD-SNP, PolyPhen-1, PolyPhen-2, SIFT, SNAP, and PANTHER identified two, three, and four mutations as deleterious, respectively. PANTHER categorized five mutations as having an unknown effect (Table 4). The MAGPIE website indicated that all the mutations were pathogenic mutations (Table 5). The iStable server integrates the predictions from iMutant, MUpro, and iStable. Specifically, iMutant, MUpro, and iStable predicted one, three and four mutations, respectively, that would result in reduced stability of the protein (refer to Table 6).

#### 3.3.3. Biophysical Characterization and Conservation Analysis

**Figure 2:**
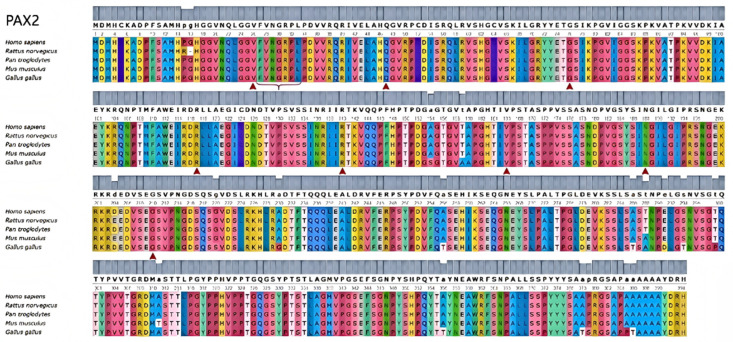
Comparative analysis of mutations in *PAX2*-related genes. Conservation analysis of pathogenic mutation amino acid positions in PAX2 via UGENE.

**Figure 3:**
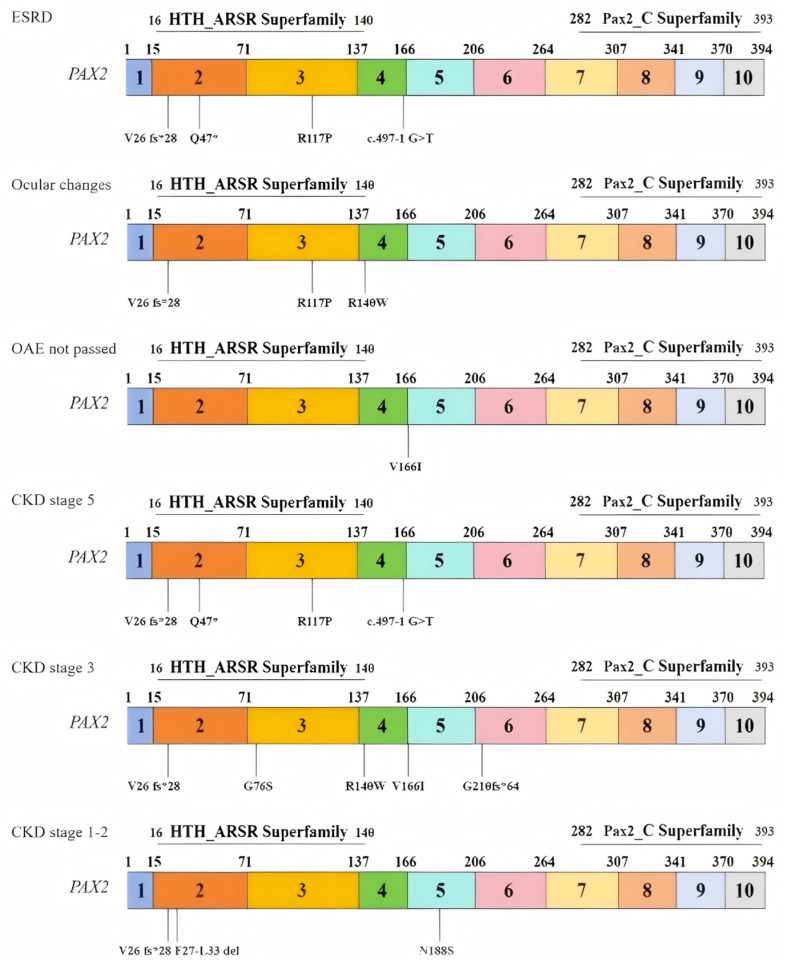
Relationships between the distributions of variants and patient phenotypes.

To investigate changes in the biophysical properties of amino acids via multiple sequence alignment (MSA), the Align GVGD tool (http://agvgd.hci.utah.edu/, v2006) was used. Among the 5 mutations examined, 2 were classified as class C65, indicating a greater likelihood of causing damage (refer to Table 6). Taking into account the results obtained from all the tools, two specific mutants, namely, R117P and R140W, were selected for further analysis (refer to Table 7). Each identified mutation was subsequently analyzed via UGENE software (UniPro, Guangzhou, China, v52.1) to assess its evolutionary conservation. Most of them demonstrated a high degree of conservation (Figure 2). We also outlined the relationship between the mutation domain and clinical phenotypes. The variants were distributed mainly in the domain of the HTH_ARSR superfamily of *PAX2* (Figure 3).

Distribution of variants in exons and domains on the basis of three clinical phenotypes: (1) end-stage renal disease (ESRD); (2) ocular changes; (3) OAEs not passed; and (3) distribution of variants in exons and domains on the basis of three stages of CKD: (1) stage 5; (2) stage 3; and (3) stages 1–2.

## 4. Discussion

We reported 14 children from a single center who had diseases associated with heterozygous mutations in the *PAX2* gene. Among them, five children had reached ESRD, and thirteen children had renal involvement (92%). In terms of extrarenal manifestations, three out of five children (60%) had eye changes, whereas one out of seven children (14%) had abnormal hearing test results. Additionally, two patients presented with scoliosis, which is a novel finding not previously reported. We identified five new mutants that expand the spectrum of *PAX2* variants. For the five missense mutations, we utilized bioinformatics methods to predict their pathogenicity.

Mutation records in reports in the relevant literature, such as the LOVD (www.lovd.nl/PAX2, accessed on 22 February 2025) and ClinVar (https://www.ncbi.nlm.nih.gov, accessed on 22 February 2025) databases, suggest that the hotspot mutation of *PAX2* is located at the 76th guanylate [15]. This position is situated in the second exon, specifically in the DNA pairing domain, leading to frameshift mutations, resulting in changes in amino acid translation and a decrease in the activity and function of the *PAX2* protein. However, even individuals with the same genotypic variant, such as c.76dupG, exhibit varying degrees of renal function damage and progress to ESRD at different ages. This has been confirmed in multiple reports [16,17], including studies involving identical twins with *PAX2* mutations [18] and our own study involving four children with c.76dupG. Previous studies have concluded that establishing a direct correlation between the clinical phenotype and genotype of *PAX2* mutations is challenging. Regardless of the type or location of the mutation, most mutations result in highly variable phenotypes [6,15,17]. Some studies suggest that this variability may be attributed to haploinsufficiency of *PAX2* heterozygous mutations [19], as well as epigenetic modifications or environmental factors [20].

PAX2 is a critical transcription factor in renal development, and its dosage is essential for proper organogenesis [19]. Previous studies, including animal models, have shown that insufficient PAX2 expression can impair nephron formation and kidney growth, providing a biological basis for the congenital anomalies observed in patients with *PAX2* mutations [21]. However, our clinical findings, consistent with previous literature, indicate marked variability in renal and extrarenal phenotypes even among patients with the same mutation, which may be related to genetic background, haploinsufficiency, and possible environmental or epigenetic modifiers [22,23,24]. The progressive decline of renal function with age in some patients may reflect the cumulative impact of developmental defects and secondary nephron overload, as previously hypothesized, but further mechanistic studies are needed to clarify these processes in humans.

Our study used PredictSNP, MAGPIE, and Align GVGD to provide more accurate disease-related mutation predictions. Combining the results of all the model predictions, we strongly predicted two specific mutants, R117P and R140W, which are highly correlated with disease pathogenicity and are likely to have potential damage far exceeding that of other gene mutants. Patients carrying these two mutant genes should also have more severe clinical manifestations.

Among the five cases of missense mutations identified in this study, R117P is the only mutation that progresses to ESRD, suggesting that R117P may have increased pathogenicity. Patients carrying R117P mutations were observed to have more severe clinical symptoms, poorer disease conditions, and multiple concurrent manifestations, such as papilledema and short stature. These findings are consistent with our predictions and strongly suggest that R117P may play a significant role in the development of *PAX2*-related diseases. Although patients carrying R140W do not progress to ESRD, they also present symptoms of papilledema and significantly elevated serum creatinine (sCr) levels. Additionally, their eGFRs are relatively low, indicating severe renal damage and a relatively poor prognosis. These clinical data are highly consistent with our predictions and suggest that R140W plays an important role in the development of *PAX2*-related diseases.

In addition, we used MAGPIE to conduct an in-depth analysis of all the mutations, and all the mutations except for p.F27-L33 del and N188S presented high pathogenicity scores. Notably, although the sCr and eGFR of the patient with the p.F27-L33 del mutation deviated from the normal range, the degree of deviation was small, indicating a good prognosis. The patients had stage 2 chronic kidney disease (CKD), and their degree of disease progression was lower than that of other patients, which is consistent with our prediction results.

The low pathogenicity of N188S was confirmed in patient number 13, who had CKD stage 1. However, this patient also had hydronephrosis and kidney stones, which could be linked to a family history of kidney stones or other unknown pathogenic factors. Further investigation is needed. In this study, the disease progression and severity in patients with p.V26 fs28 gene mutations were inconsistent. Although previous studies have reported its potential pathogenicity [6], we believe that carrying the p.V26 fs28 mutation may be just one of many pathogenic factors, and there may be a hidden pathogenesis that has not yet been revealed, which needs further research in the future.

Compared to previous reports, our study provides several new insights: (1) Identification of five previously unreported *PAX2* variants in Chinese children, thereby expanding the global *PAX2* mutation spectrum; (2) Description of rare extrarenal phenotypes such as scoliosis, which have not been previously associated with *PAX2* mutations; (3) Detailed genotype–phenotype analysis in a relatively large cohort from a single center, highlighting the variable expressivity and incomplete penetrance of *PAX2*-related disorders in a Chinese pediatric population. These findings enrich the current understanding of *PAX2*-related disease manifestations and may have implications for genetic counseling and clinical management in this population.

## 5. Conclusions

In summary, this investigation underscores the intricate genetic composition of *PAX2* and emphasizes the significance of early identification and intervention. Consequently, annual physical examinations are essential for the detection of kidney diseases, while it is imperative that children exhibiting idiopathic renal failure and bilateral renal agenesis undergo genetic testing, thereby preventing the administration of unnecessary hormonal treatments, and kidney protective treatment should be initiated early.

## Figures and Tables

**Table 1:** General condition and renal manifestations.

Patients	1	2	3	4	5	6	7	8	9	10	11	12	13	14
Group	ESRD					un ESRD								
*General characteristics*														
Gender	F	M	F	F	F	M	F	F	M	M	M	F	F	F
Onset age (years)	0.2	9.9	1	6	8	12	9	7	5.9	prenatal	12.8	6.5	prenatal	NB
ESRD/age	Y/11	Y/10	Y/1	Y/6	Y/8	N	N	N	N	N	N	N	N	N
Visit age	11.7	10	7	6	8	12	12.1	8.4	5.9	0.5	12.8	13.5	0.1	4
Growth retardation	-	-	+	-	-	-	+	-	-	-	-	-	-	-
sCr (umol/L)	513	1089	350	1080	381	206	141	68	61	61	207	87	21	135
eGFR (mL/min/1.73 m^2^)	10	5.5	15	4	12	41.03	49.78	73.7	76.8	43.2	36.8	71	104	54.35
CKD stage	5	5	5	5	5	3	3	2	2	3	3	2	1	3
*Urine testing indicators*														
PU	+/NP	+/NP	+/NP	+/NP	+/NP	+	+	-	-	+	+/NP	-	-	+
HU/HBC per HB	-	+/4	-	-		-	-	-	-	+/5	-	-	-	-
24 h UP (mg)	1280	1585	1697	916	2427	2272	563	172	66	NA	4119	160	NA	682
ACR (mg/gCr)	1615	7337	1804	2358	1958	1615	466	118	32	2589	2902	15	84	1180
α1MG/CR (mg/gCr)	269	197	225	245	315	19	269	8	7	269	80	11	21	124
*Urinary system Image Assessment*						13/14 (92%) Ultrasound describes small kidney size (<−2 SD)								
Kidney size (US)														
Age	11.7	10	7	6	8	12	12.1	8.4	5.9	0.5	12.8	13.5	0.1	4
L (cm)	5.1 × 2.8	6.5 × 3.2	5.2 × 2.3	5.4 × 1.9	−2SD	8.3 × 4.5	4.6 × 2.3	6.7 × 2.5	4.8	4.2 × 2.0	8.3 × 3.8	9.6 × 3.1	4.8 × 2.1	−2SD
R (cm)	4.7 × 2.6	Absent	4.6 × 2.1	5.1 × 2	−2SD	7.8 × 3.3	5.8 × 2.7	7.2 × 2.3	6.7	4.3 × 1.9	8.1 × 4.0	8.8 × 3.6	5.0 × 1.6	−2SD
Cysts/Location	-	-	+/R/S	-	+/B/M	+/B/M	+/L/S	-	-	+/B/M	-	-	-	-
Additional	-	-	-	-	-	-	-	Hydronephrosis	-	-	-	-	Stone, Hydronephrosis	-

PU, proteinuria. NP, nephrotic-range proteinuria (≥50 mg/kg/day). HU, hematuria, urine HBC ≥ 3/Hp. 24 h UP, 24-h urine protein. ACR, urine albumin/urine creatinine. α1MG/CR, urine α1 microglobulin/urine creatinine. US, ultrasonic testing. Cysts/location, L/R/B-left/right/bilateral, S/M-single/multiple. NA, not available.

**Table 2:** Extrarenal manifestations.

	1	2	3	4	5	6	7	8	9	10	11	12	13	14
Group	ESRD				un ESRD									
*Extrarenal manifestations*														
Eyes	NA	NA	Papill-edema	NA	NA	NA	NA	-	-	NA	Papill-edema	Morning glory anomaly	NA	NA
Ears	-	NA	-	NA	NA	-	-	NA	NA	NA	-	-	NA	OAE not passed
Neurological system	-	-	-	-	-	-	-	-	-	-	-	-	-	-
Bones	-	-	-	-	-	-	Scoliosis	-	-	-	-	Scoliosis	-	-

OAE, otoacoustic emission. NA, not available.

**Table 3:** *PAX2* gene variants.

	1	2	3	4	5	6	7	8	9	10	11	12	13	14
Group	ESRD				un ESRD									
Nucleotide change	c.139 C>T	c.76 dupG	c.350 G>C	c.497-1 G>T	EXON:1-10del	c.226 G>A	c.76 dupG	c.76 dupG	c.78–c.98 del	c.629 delG	c.418 C>T	c.76 dupG:	c.563 A>G	c.496 G>A
Amino change	p.Q47*	p.V26 fs*28	p.R117P	-	-	p.G76S	p.V26 fs*28	p.V26 fs*28	p.F27-L33 del	p.G210 fs*64	p.R140W	p.V26 fs*28	p.N188S	p.V166I
Pathoge-nicity	P	P	P	P	P	VUS	P	P	VUS	P	LP	P	LP	LP
EXON	2	2	3	-	1-10	3	2	2	2	6	4	2	5	4
Type	N	F	M	S	CNV	M	F	F	I	F	M	F	M	M
Zygosity/ segregation	Het/D	Het/D	Het/D	Het/D	Het/D	Het/M	Het/D	Het/D	Het/D	Het/D	Het/D	Het/F	Het/F	Het/F
Family history	-	-	-	-	-	-	-	-	-	-	Father uremia	Father uremia	kidney stones	-
Reported	-	+	+	+	-	+	+	+	-	-	+	+	+	-

Mutation type: N, nonsense mutation; F, frameshift mutation; I, inframe mutation; S, splice variation; CNV, copy number variation. Zygosity/segregation: het, heterozygous; F/M/D, father/mother/de.

**Table 4:** Deleteriousness prediction of *PAX2* (NP_000269.3) mutations using the PredictSNP server.

Serial Number	Accession Number	Amino Acid Change	Predict SNP	MAPP	PhD-SNP	PolyPhen-1	PolyPhen-2	SIFT	SNAP
1	NP_000269.3	V166I	N	N	N	N	N	N	N
2	NP_000269.3	G76S	D	D	D	D	D	D	D
3	NP_000269.3	R117P	D	D	D	D	D	D	D
4	NP_000269.3	R140W	D	D	D	D	D	D	D
5	NP_000269.3	N188S	N	N	D	N	N	N	N

D, deleterious; N, neutral.

**Table 5:** The pathogenicity of *PAX2* (NP_000269.3) mutations was predicted via the MAGPIE website.

Serial Number	Accession Number	Amino Acid Change	Nucleotide Change	Chr	Start	End	MAGPIE Prediction
1	NP_000269.3	V166I	c.496G>A	10	100779583	100779583	0.993759408
2	NP_000269.3	G76S	c.226G>A	10	100750707	100750707	0.947722773
3	NP_000269.3	R117P	c.350G>C	10	100750831	100750831	0.904887777
4	NP_000269.3	R140W	c.418C>T	10	100749841	100749841	0.999467114
5	NP_000269.3	N188S	c.563A>G	10	100781312	100781312	0.036130005
6	NP_000269.3	p.Q47*	c.139C>T	10	100779505	100779505	0.94064358
7	NP_000269.3	p.V26 fs*28	c.76dupG	10	100749778	100749778	0.968490245
8	NP_000269.3	p.F27-L33 del	c.78-c.98del	10	100749783	100749803	0.067596214
9	NP_000269.3	p.G210 fs*64	c.629delG	10	100806442	100806442	0.97295275
10	NP_000269.3	-	c.497-1G>T	10	100781245	100781245	−1 (invalid for splicing)

**Table 6:** Change in stability prediction via the iStable server and biophysical characterization via the Align GVGD server.

Serial Number	Accession Number	Amino Acid Change	i-Mutant2.0 SEQ	DDG	MUpro	Conf. Score	iStable	Conf. Score	GV	GD	Prediction
1	NP_000269.3	V166I	D	−0.52	D	−1	D	0.708591	0	29.61	Class C25
2	NP_000269.3	G76S	D	−1.33	I	0.084420636	I	0.506123	0	55.27	Class C55
3	NP_000269.3	R117P	D	−0.86	D	−0.46061846	D	0.751511	0	102.71	Class C65
4	NP_000269.3	R140W	D	−0.35	D	−0.7478608	D	0.828271	0	101.29	Class C65
5	NP_000269.3	N188S	D	−0.65	I	0.17276469	I	0.654345	0	46.24	Class C45

D, decrease; I, increase.

**Table 7:** Phenotypic effect prediction of pathogenic *PAX2* mutations via the SNPeffect server.

Serial Number	Mutation	TANGO	WALTZ	LIMBO	FoldX
1	R117P	does not affect the aggregation tendency of your protein	does not affect the amyloid propensity of your protein	increases the chaperone binding tendency of your protein	reduces the protein stability
5	R140W	does not affect the aggregation tendency of your protein	does not affect the amyloid propensity of your protein	does not affect the chaperone binding tendency of your protein	has no effect on the protein stability

## Data Availability

The dataset used for the current study is available from the corresponding author on reasonable request.

## References

[1] Terzić J., Muller C., Gajović S., Saraga-Babić M. (1998). Expression of PAX2 gene during human development. Int. J. Dev. Biol..

[2] Torres M., Gómez-Pardo E., Dressler G.R., Gruss P. (1995). Pax-2 controls multiple steps of urogenital development. Development.

[3] Thompson B., Davidson E.A., Liu W., Nebert D.W., Bruford E.A., Zhao H., Dermitzakis E.T., Thompson D.C., Vasiliou V. (2021). Overview of PAX gene family: Analysis of human tissue-specific variant expression and involvement in human disease. Hum. Genet..

[4] Harshman L.A., Brophy P.D. (2012). PAX2 in human kidney malformations and disease. Pediatr. Nephrol..

[5] Muntean C., Chirtes C., Baczoni B., Banescu C. (2023). PAX2 Gene Mutation in Pediatric Renal Disorders-A Narrative Review. Int. J. Mol. Sci..

[6] Bower M., Salomon R., Allanson J., Antignac C., Benedicenti F., Benetti E., Binenbaum G., Jensen U.B., Cochat P., DeCramer S. (2012). Update of PAX2 mutations in renal coloboma syndrome and establishment of a locus-specific database. Hum. Mutat..

[7] Weber S. (2012). Novel genetic aspects of congenital anomalies of kidney and urinary tract. Curr. Opin. Pediatr..

[8] Harrison S.M., Biesecker L.G., Rehm H.L. (2019). Overview of Specifications to the ACMG/AMP Variant Interpretation Guidelines. Curr. Protoc. Hum. Genet..

[9] Bendl J., Stourac J., Salanda O., Pavelka A., Wieben E.D., Zendulka J., Brezovsky J., Damborsky J. (2014). PredictSNP: Robust and accurate consensus classifier for prediction of disease-related mutations. PLoS Comput. Biol..

[10] Chen C.W., Lin J., Chu Y.W. (2013). iStable: Off-the-shelf predictor integration for predicting protein stability changes. BMC Bioinform..

[11] Tavtigian S.V., Deffenbaugh A.M., Yin L., Judkins T., Scholl T., Samollow P.B., de Silva D., Zharkikh A., Thomas A. (2006). Comprehensive statistical study of 452 BRCA1 missense substitutions with classification of eight recurrent substitutions as neutral. J. Med. Genet..

[12] Glaser F., Pupko T., Paz I., Bell R.E., Bechor-Shental D., Martz E., Ben-Tal N. (2003). ConSurf: Identification of functional regions in proteins by surface-mapping of phylogenetic information. Bioinformatics.

[13] Liu Y., Zhang T., You N., Wu S., Shen N. (2024). MAGPIE: Accurate pathogenic prediction for multiple variant types using machine learning approach. Genome Med..

[14] Obrycki Ł., Sarnecki J., Lichosik M., Sopińska M., Placzyńska M., Stańczyk M., Mirecka J., Wasilewska A., Michalski M., Lewandowska W. (2022). Kidney length normative values in children aged 0–19 years—A multicenter study. Pediatr. Nephrol..

[15] Yang X., Li Y., Fang Y., Shi H., Xiang T., Liu J., Liu J., Tang X., Fang X., Chen J. (2021). Phenotypic spectrum and genetics of PAX2-related disorder in the Chinese cohort. BMC Med. Genom..

[16] Zhang L., Zhai S.-B., Zhao L.-Y., Zhang Y., Sun B.-C., Ma Q.-S. (2018). New PAX2 heterozygous mutation in a child with chronic kidney disease: A case report and review of the literature. BMC Nephrol..

[17] Deng H., Zhang Y., Xiao H., Yao Y., Liu X., Su B., Zhang H., Xu K., Wang S., Wang F. (2019). Diverse phenotypes in children with PAX2-related disorder. Mol. Genet. Genom. Med..

[18] Iatropoulos P., Daina E., Mele C., Maranta R., Remuzzi G., Noris M. (2012). Discordant phenotype in monozygotic twins with renal coloboma syndrome and a PAX2 mutation. Pediatr. Nephrol..

[19] Porteous S., Torban E., Cho N.-P., Cunliffe H., Chua L., McNoe L., Ward T., Souza C., Gus P., Giugliani R. (2000). Primary renal hypoplasia in humans and mice with PAX2 mutations: Evidence of increased apoptosis in fetal kidneys of Pax2(1Neu) +/− mutant mice. Hum. Mol. Genet..

[20] Hilliard S.A., El-Dahr S.S. (2016). Epigenetics mechanisms in renal development. Pediatr. Nephrol..

[21] Quinlan J., Lemire M., Hudson T., Qu H., Benjamin A., Roy A., Pascuet E., Goodyer M., Raju C., Zhang Z. (2007). A common variant of the PAX2 gene is associated with reduced newborn kidney size. J. Am. Soc. Nephrol..

[22] Min J., Luo Y., Fu Q., Sun X., Mi L., Shen Y., Wang H. (2025). Clinical spectrum, genetics and management insights of PAX2-related disorder in nine children. Eur. J. Med. Res..

[23] Negrisolo S., Benetti E. (2023). PAX2 and CAKUT Phenotypes: Report on Two New Variants and a Review of Mutations from the Leiden Open Variation Database. Int. J. Mol. Sci..

[24] Xiong H.Y., Shi Y.Q., Zhong C., Yang Q., Zhang G., Yang H., Wu D., Chen Y., Li Q., Wang M. (2022). Detection of De Novo PAX2 Variants and Phenotypes in Chinese Population: A Single-Center Study. Front. Genet..

